# The Long Term Economic Impact of Severe Obstetric Complications for Women and Their Children in Burkina Faso

**DOI:** 10.1371/journal.pone.0080010

**Published:** 2013-11-05

**Authors:** Patrick G. C. Ilboudo, Steve Russell, Ben D’Exelle

**Affiliations:** 1 Agence de Formation, de Recherche et d’Expertise en Santé pour l’Afrique (AFRICSanté), Bobo-Dioulasso, Burkina Faso; 2 Department of Community Medicine, University of Oslo, Oslo, Norway; 3 School of International Development, University of East Anglia, Norwich, United Kingdom; UCL Institute of Child Health, University College London, United Kingdom

## Abstract

This study investigates the long term economic impact of severe obstetric complications for women and their children in Burkina Faso, focusing on measures of food security, expenditures and related quality of life measures. It uses a hospital based cohort, first visited in 2004/2005 and followed up four years later. This cohort of 1014 women consisted of two main groups of comparison: 677 women who had an uncomplicated delivery and 337 women who experienced a severe obstetric complication which would have almost certainly caused death had they not received hospital care (labelled a “near miss” event). To analyze the impact of such near miss events as well as the possible interaction with the pregnancy outcome, we compared household and individual level indicators between women without a near miss event and women with a near miss event who either had a live birth, a perinatal death or an early pregnancy loss. We used propensity score matching to remove initial selection bias. Although we found limited effects for the whole group of near miss women, the results indicated negative impacts: a) for near miss women with a live birth, on child development and education, on relatively expensive food consumption and on women’s quality of life; b) for near miss women with perinatal death, on relatively expensive foods consumption and children’s education and c) for near miss women who had an early pregnancy loss, on overall food security. Our results showed that severe obstetric complications have long lasting consequences for different groups of women and their children and highlighted the need for carefully targeted interventions.

## Introduction

The reduction of maternal mortality has been high on the international development agenda through the Millennium Development Goal 5 [1]. Consequently, several policy interventions such as safe motherhood initiatives have been implemented worldwide [2]. Maternal mortality ratio reductions have been reported around the world [3]. In spite of this, each year, more than 270,000 women still die while in pregnancy or childbirth, with a large part of these deaths taking place in Sub-Saharan Africa [4]. More notable for this study, millions of women who survive severe obstetric complications in developing countries experience high levels of physical and mental morbidity [5-11]. The economic costs incurred by poor households due to these complications often lead to a significant economic burden and processes of impoverishment. 

Various studies have shown that hospitalization during childbirth and treatment for near miss complications can lead to very high expenses for households, especially in less developed countries where user fees are charged and insurance mechanisms to protect against catastrophic healthcare expenditures, defined as any payment exceeding 40% of household capacity to pay [12], are lacking [13,14]. Coping strategies to mobilise resources to pay for treatment expenses that are catastrophic often force households to reduce consumption of essential food and other goods, deplete savings, sell assets, withdraw their children from school [15] and incur high levels of debt [13,14,16], eventually keeping or pushing households into poverty [12,14,16-21].

Negative impacts may not only compromise actual earnings and living conditions of vulnerable households but could also have long lasting consequences for the household economy. This may be a realistic scenario for many households living in low income settings such as Burkina Faso. Quantitative research conducted in this country has demonstrated that women who survive severe obstetric complications experience ongoing morbidity including postpartum incontinence, fistulae and postpartum depression and anxiety [7], with ongoing treatment costs and economic repercussions. Qualitative findings have also highlighted the long term consequences of maternal near miss events including loss and disruptions in bodily integrity, ongoing illness, loss of strength, stamina and disruption of social identity and social stability [22]. 

However, there is only limited quantitative evidence about the long term effects of severe (near miss) obstetric complications on the economic and social well-being of women and their children. A good opportunity to measure such long-term effects arose in 2008/2009 in a follow-up study on the long term health and socio-economic effects of near miss events. This paper presents a quantitative analysis of economic data from this longitudinal study, running from 2004/2005 to 2008/2009.

## Methods

### Ethics Statement

Both the first and the follow-up studies were accepted by the ethics committees in Burkina Faso (Health Research Ethics Committee) and the United Kingdom (Ethics Committee of the London School of Hygiene & Tropical Medicine - LSHTM). 

Written informed consent was obtained from all participants before participation in interviews. Where participants had difficulty reading in French, interviewers read out the consent form in participants’ preferred local language to help them understand the study. Once they understood the study, their written consent was then obtained (either they signed or applied their fingerprint on the consent form). All named ethics committee specifically approved this study.

### Study site and sampling

The study was carried out in Burkina Faso, a West-African country that has limited resources, poor health outcomes and a weak health system [23], with 46% of its inhabitants living under the poverty line [24]. Maternal mortality is high with a rate of 700 per 100,000 births [25] and only 54% of births are attended by skilled health staff [25]. Large disparities exist in skilled birth attendance ratios between rural and urban populations. Referral systems are weak and in case of emergency, women are obliged to travel long distances to reach hospitals. Since 2006, a national subsidy policy for normal deliveries and emergency obstetric care has been applied in Burkina Faso. Nevertheless, user fees are still charged for delivery services at government hospitals as the policy does not abolish them. Before 2006, the average fee for a normal delivery was CFA27,245 equivalent to US$59; a fee level beyond the monthly budget of a poor household in Burkina Faso. Complicated deliveries requiring surgery or other treatment would incur much higher costs [14].

This study is built on an existing cohort of 1014 women from seven rural and urban referral hospital facilities in Burkina Faso. These women were prospectively recruited in the seven hospital facilities between December 2004 and March 2005. The facilities included the country’s two referral teaching hospitals (located in Bobo-Dioulasso and Ouagadougou), two regional hospitals (located in Dédougou and Tenkodogo) and three district hospitals with surgical capacity (located in Houndé, Nouna and Ouagadougou). The cohort consisted of two groups of comparison: 677 women who had an uncomplicated delivery and 337 women who experienced a severe obstetric complication that would have almost certainly caused death had they not received hospital care (labelled a “near miss” event). The group of women who experienced a severe obstetric complication (n=337) fell into three sub-groups: those who had a live birth (n=199), those who experienced a perinatal death (n=64) and those who had an early pregnancy loss (n=74). The diagnosis of a complicated delivery was based on clinical signs and symptoms of complication as well as case management procedures. Women with uncomplicated deliveries were defined as those who had no documented signs of obstetric complications and who vaginally delivered a live born infant at term. 

Data for physical, mental, economic and social indicators were collected from women’s medical records and at-home visits within a week after discharge and at 3 months, 6 months and 12 months. The first interview included questions on demographic, personal and socio-economic factors before the delivery. Data from the first study could be accessed through a request to the Initiative for Maternal Mortality Programme Assessment – Immpact website: http://www.immpact-international.org – University of Aberdeen (UK).

### The follow-up study

The follow-up study was done over a period of four months in 2008 and 2009. It managed to track 711 women i.e. 70% of the 2004/2005 cohort. Each woman was located and invited to participate in the study with her husband and/or the head of household. Whenever possible, interviewers who participated in the first study were recruited to work on the follow-up. Following informed consent, women were invited to provide answers to a structured interview on their current socio-demographic characteristics, health, economic and social well-being including their perceived health. Men and other key family members (if the husband was not the head of the household) were also invited to answer questions on income, consumption and assets in separate interviews. Data from the follow-up study could be accessed through a request to the Economic and Social Research Council – ESRC: website http://www.esrc.ac.uk – (UK).

### Statistical analysis

To make sensible claims about the impact of a near miss event, we need to find a suitable ‘counterfactual’ to the exposure of such event. In other words, it would have been ideal to know what would have happened, had women with life-threatening delivery complications not been exposed to a near miss event. This is obviously technically impossible as women cannot at the same time be affected and not affected by near miss events. To solve this problem, impact studies compare women exposed to such an event (treatment group) with women not exposed (control group). However, when comparing outcome indicators between women who experienced obstetric complications and women who did not experience such complications, as a measurement of impact, one should be aware that such a comparison may suffer from selection biases. To illustrate this, imagine we are interested in measuring the effect of near miss events on women’s well-being. For this, we compare well-being indicators of women who suffered a near miss event and those who had an uncomplicated delivery. However, differences in well-being indicators may be biased by socio-economic differences that already existed between the two groups, which in this setting is more likely because our sample is hospital-based: women from wealthier households tend to go to hospital irrespective of expecting complications [26-30], while women from poorer households only go to hospital when expecting complications or when unexpected complications suddenly arise. Women with complications who were selected into our sample may, on average, be poorer than women without complications. 

It is therefore necessary to adjust samples so that the likelihood of being exposed to a near miss event conditional on important socio-economic characteristics (e.g. wealth) is made similar across the treatment and comparison groups. To achieve this, we resorted to propensity score matching techniques (PSM) [31]. The basic idea of PSM is to match women with uncomplicated deliveries to women with near miss complications who are similar in all observable characteristics that might be correlated with the likelihood of having a near-miss event. Any remaining differences in outcomes of both matched groups can then be attributed to the near miss complication only. 

PSM consists of two phases. In the first step, we ran a probit regression, pooling together near miss and uncomplicated delivery women and we estimated the probability that a woman in the sample belonged to the near miss group, controlling for relevant confounding variables. The empirical literature showed us that richer women, women in urban areas, higher educated women, more empowered women or women who had not had many children before were more likely to deliver in a hospital in absence of any complication, whereas poorer women, women in rural areas, lower educated women, women who had had more children, less empowered women only did so if it was really necessary [32-40]. We therefore added variables such as education and residence in the probit regression. We also controlled if women had an economic activity, the number of live births still living with them, gravidity, marital status and a decision-making power dummy equal to 1 if the woman participated in the decision-making on household expenditures. To control for household wealth we added an asset index based on a principal component analysis of wealth variables such as possession of a radio, car, bicycle, fridge etc.

In the second step, women with a near miss complication were matched with women with an uncomplicated delivery and who had similar propensity scores. We used a ‘stratified’ matching algorithm, which matched observations between near miss and uncomplicated delivery women within different strata of propensity scores in order to balance the distribution of the observed predictor variables of near miss between both groups. To ease interpretation of the results, we re-coded all variables so that a negative sign reads as a negative impact. Out of the 711 women we recontacted, complete information was available for only 698 women and all analyses were performed on stata version 11.2 based on these 698 observations.

### Outcome indicators

The main outcome variables at the household level used for the analysis were: weekly consumption of a selected relatively expensive food items, estimated income approximated via household expenditures on various items, expenditures on education and index child development measured through their height and weight. The index child is defined as the 2004/5 surviving child of women who had a severe obstetric complication (near miss). The child’s weight was measured with the use of a calibrated balance, with a registered record in a standing position at nearly 100 grams. The child’s height was measured without wearing shoes and standing back against a vertical wall by the use of a tape. The analysis of the weight and height consisted of a comparison of the index child weight and height with, that of the child of women who had had an uncomplicated delivery in 2004/5. We also used food insecurity as measured by the Maxwell food insecurity grid [41]. Each question on the Maxwell grid was weighted from 1 to 4, based on the in-depth knowledge of the research team of consumption habits and food coping strategies in Burkina Faso. We then added up the weights together to produce a food insecurity index with a high score indicating food security at the household level. We also used the WHO quality of life assessment grid that measures the post-partum subjective quality of life [42]. Each question was again given a weight ranging from 1 for total unsatisfaction to 3, meaning entire satisfaction. We added up the scores to produce an index of an overall perceived well-being, a high score meaning more satisfaction for one’s quality of life. 

## Results

This section is subdivided into 5 sub-sections. The first sub-section describes the prevalence of loss to follow-up and the characteristics of the longitudinal sample in 2004/2005 and in 2008/2009. The second sub-section depicts the prevalence of poverty in the sample of households in 2004/2005. The third sub-section presents the 2004/5 delivery cost borne by the near miss and uncomplicated delivery households. The fourth sub-section presents the results of the aggregated analysis, comparing the whole group of near miss women with the control group. The final sub-section presents the disaggregated analysis of the comparison of each type of near miss women with the uncomplicated delivery group.

### Loss to follow-up and characteristics of the longitudinal sample in 2004/2005 and in 2008/2009

Table **1** and Table **2** respectively present the prevalence of loss to follow-up and the most important characteristics of the sample. Only 70% of the original sample was reached in 2009 (Table **1**). Among women who were lost to follow-up, the greatest loss was for the near miss women who had lost their child (42%) followed by near miss women with live birth (34%). Near miss women with live birth were the most represented in our sample of near miss women in 2008/2009 with a percentage of 59% (Table **2**). While in 2004/5, only 43% of the women were economically active through formal or informal employment, this percentage was substantially higher (60%) four years later. Most of the women in the sample remained married (84%) in 2009 even if we noticed an overall decrease in the percentage of women married in a monogamous arrangement (71% to 63%). Women with near miss complications were more likely to be separated or divorced from their 2004/5 partners (8% versus 5%), and more likely to be widowed (2% versus 1%) compared to the uncomplicated delivery women in 2009. 

**Table 1:** Samples sizes in 2004/2005, in 2008/2009 and loss to follow-up in 2008/2009.

	**2004/2005**	**2008/2009**	
**Type of pregnancy outcome**	**N(%)**	**Follow-up (%)**	**Loss to follow-up (%)**
Near miss with perinatal death	74 (100)	43 (58)	31 (42)
Near miss with abortion	64 (100)	45 (70)	19 (30)
Near miss with live birth	199 (100)	131 (66)	68 (34)
Uncomplicated delivery	677 (100)	492 (73)	185 (27)
Total	1014 (100)	711 (70)	303 (30)

**Table 2:** Characteristics of the longitudinal sub-sample at enrolment in 2004/2005 and in 2008/2009.

		**2004/2005**			**2008/2009**		
	**Pregnancy outcome**	**All**	**Uncom^1^**	**NM^2^**	**All**	**Uncom^1^**	**NM^2^**
**Sample size**	Number of observations	698	484	214	698	484	214
**Age**	Mean (SD^3^)	26 (6.70)	26 (6.51)	26 (7.14)	-	-	-
**Distribution of marital status**	Single-never married	9%	8%	10%	6%	6%	7%
	Married monogamous	71%	73%	68%	63%	65%	60%
	Married polygamous	20%	19%	22%	21%	21%	21%
	Separated/divorced	-	-	-	7%	5%	8%
	With a new partner	-	-	-	2%	2%	2%
	Widowed	-	-	-	1%	1%	2%
**Gravidity**	Number of pregnancies (SD^3^)	3 (2.26)	3 (2.18)	3 (2.42)	4 (2.21)	4 (2.16)	4 (2.32)
**Woman’s main activity**	Housework/ unemployed	57%	56%	59%	40%	39%	44%
	Income-generating (formal or informal)	43%	44%	41%	60%	61%	56%
**Type of near miss**	Live-birth (%)	N/A	N/A	66%	N/A	N/A	59%
	Stillbirth or early neonatal death (%)	N/A	N/A	14%	N/A	N/A	20%
	Early pregnancy loss (%)	N/A	N/A	20%	N/A	N/A	21%
**Wealth**	Asset index (SD^3^)	0.02 (2.10)	0.24 (2.05)	-0.46 (2.12)	-	-	-

1=Uncomplicated delivery.

2=Near miss.

3=Standard deviation.

### Prevalence of disadvantaged households in the sample in 2004/2005

**Figure 1:**
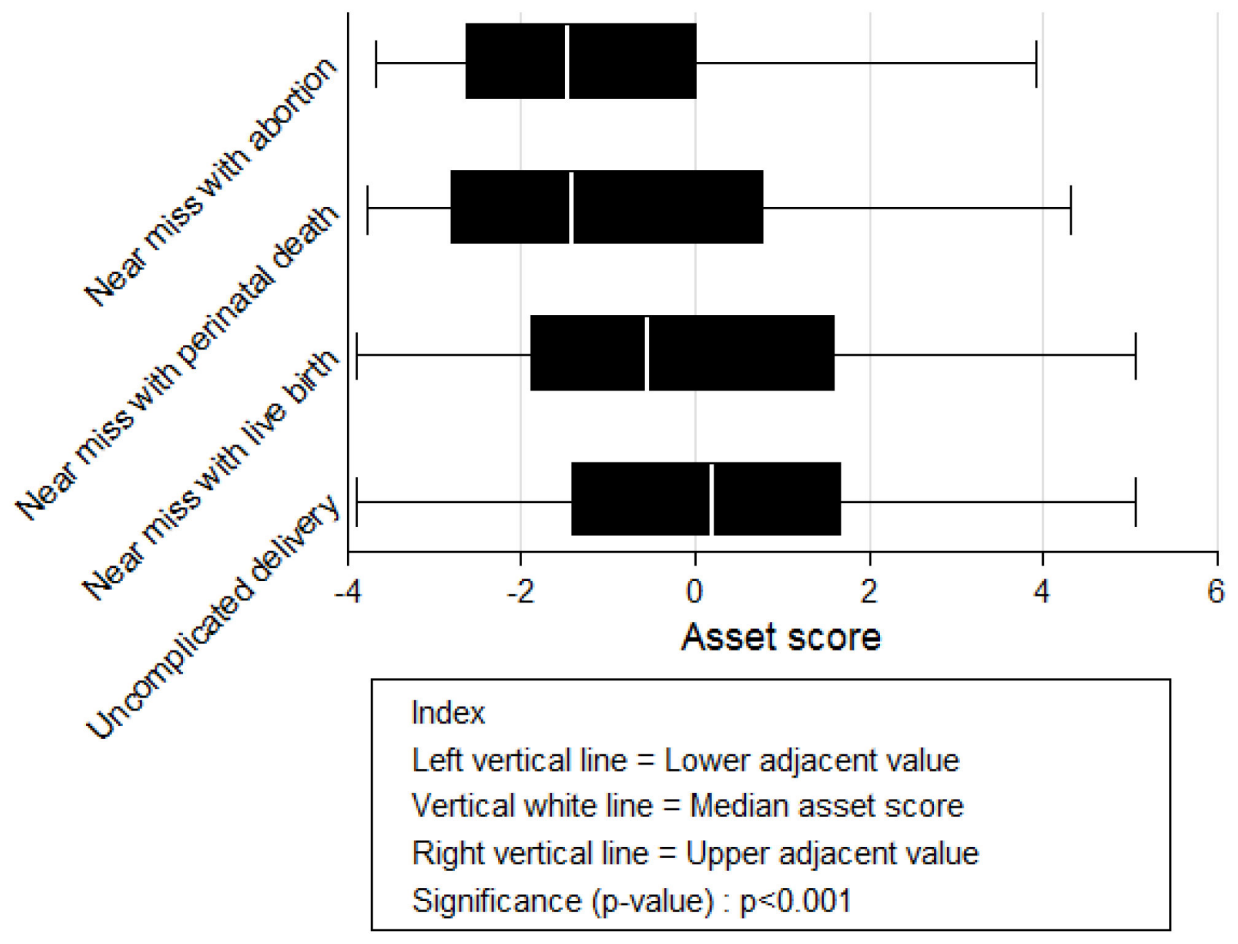
Household asset index score from principal component analysis in 2004/5.

Figure **1** shows that near miss households were poorer than uncomplicated delivery households in 2004/2005. Near miss women with abortion and those with perinatal death appeared to have similar average wealth. Moreover, near miss women with live birth have an average wealth which is higher than women with abortion and perinatal death (p< 0.001). 

### Emergency obstetric and normal delivery costs to households in 2004/5

**Figure 2:**
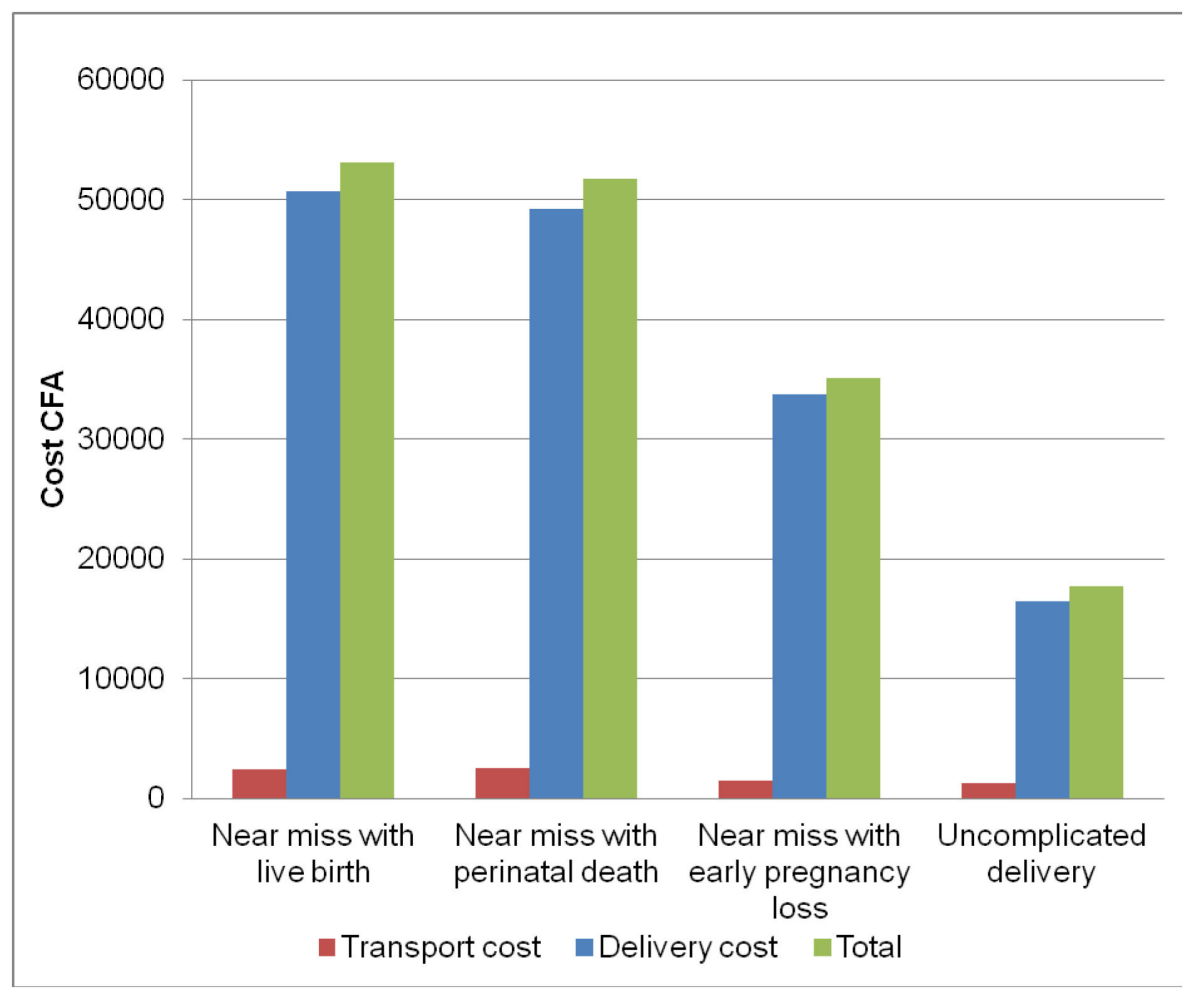
2004/5 hospital event cost by near miss group and for the uncomplicated delivery group.

Households of near miss women paid much more for hospital delivery in 2004/2005 than women with uncomplicated deliveries. Each near miss group paid more than twice the amount that the uncomplicated delivery group paid (Figure **2**). Within the near miss group, households with live birth and perinatal death incurred the highest costs for the delivery. 

### Aggregated analysis: comparison of near miss women with the control group

Table **3** presents the results of the probit regression used to estimate individual propensity scores. We found that single women, married monogamous women or either second or more than second polygamous married women were more likely to have a near miss complication. Likewise, women with a larger number of pregnancies were also more likely to have a near miss complication. Conversely, we found that women living in urban areas, women having a low number of children still living with them or those having an economic activity were less likely to have a near miss complication. 

**Table 3:** Estimations of the propensity scores between treatment and control groups.

	**Near miss vs uncomplicated delivery**	
**Variables**	**Coefficient**	**Standard error**
Economic activity	-0.198*	0.117
Married - monogamous	0.360**	0.172
Polygamous - first wife	0.457	0.471
Polygamous - second or lower wife	0.636**	0.290
Single	0.831***	0.258
Number of live births still living with mother	-0.228***	0.064
Number of pregnancies	0.185***	0.045
Modern primary education	-0.147	0.153
Modern secondary education	0.021	0.163
Alphabetisation or ‘coranique’	0.108	0.204
Living in a rural area	0.244	0.381
Living in a urban area	-0.686**	0.346
Decision making power	0.082	0.132
Asset index	-0.029	0.031
Constant	-0.350	0.403
N	655	
Pseudo R2	0.0845	
LR chi^2^	66.67	
Prob>chi^2^	0.0000	

Note: Significance levels: *** p < 0.01; ** p < 0.05; * p < 0.10.

Balancing properties satisfied for all models.

**Figure 3:**
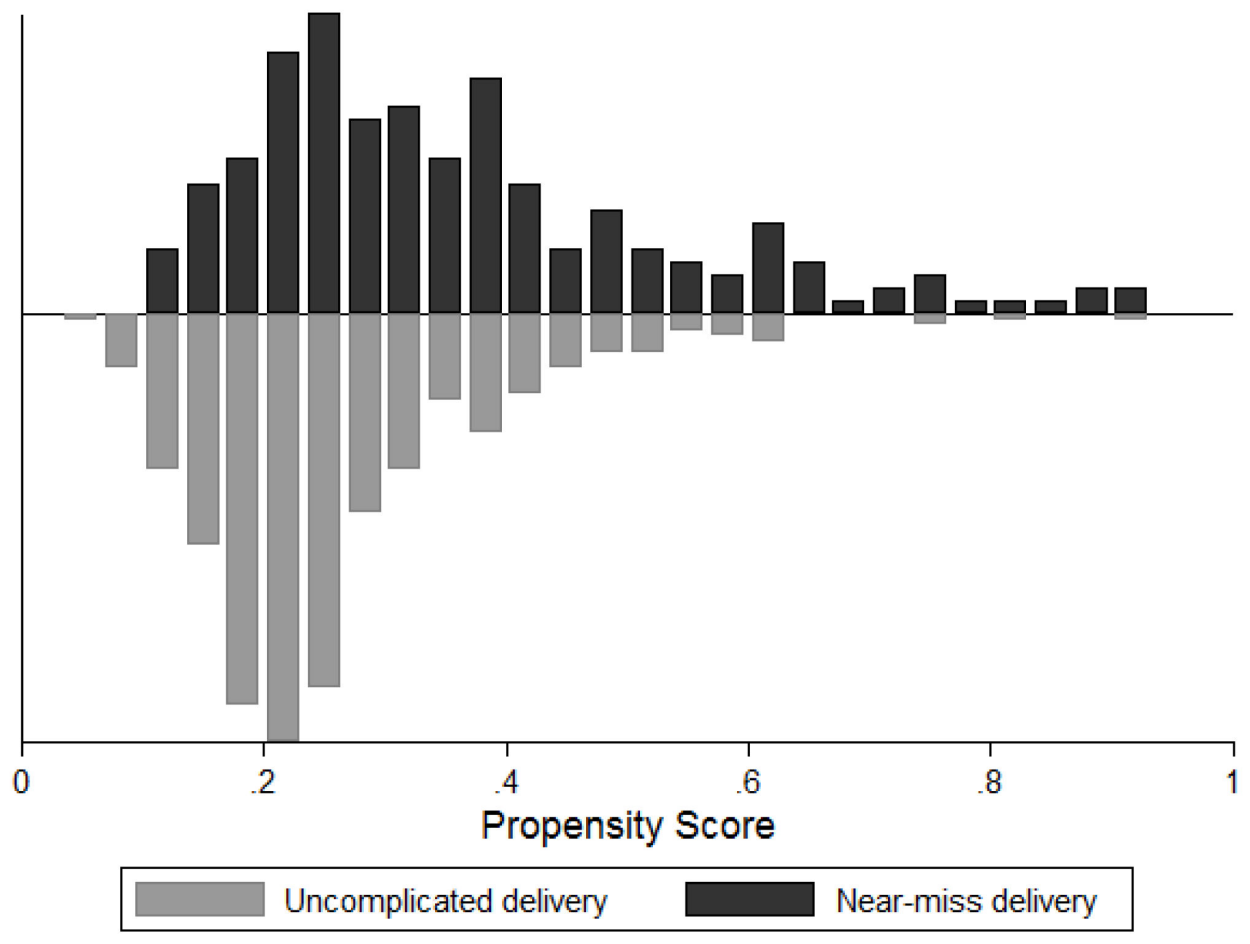
Estimated propensity scores of near miss and control groups.

The distribution of the predicted probabilities of a near miss event is presented in Figure **3**. As expected, we observed that in the group of near miss complications, propensity scores were skewed to the right compared to the uncomplicated delivery group. Table **4** further shows the balancing statistics of women’s characteristics based on standardised differences after matching. After matching, we found a satisfactory balance of the standardised differences between women’s characteristics.

**Table 4:** Standardised differences after matching on the propensity score.

**Variables**	**NM^1^**	**Un^2^**	**SD^3^**	**P-value^4^**
Economic activity	0.43	0.42	0.014	0.878
Married - monogamous	0.02	0.01	0.017	0.854
Polygamous - first wife	0.07	0.08	-0.015	0.850
Polygamous - second or lower wife	0.11	0.12	-0.036	0.663
Single	0.68	0.67	0.017	0.736
Number of live births still living with mother	2.21	2.16	0.029	0.660
Number of pregnancies	3.51	3.42	0.038	0.913
Modern primary education	0.20	0.20	-0.010	0.944
Modern secondary education	0.26	0.27	-0.022	0.811
Alphabetisation or ‘coranique’	0.09	0.09	0.006	0.904
Living in a rural area	0.18	0.17	0.056	0.242
Living in a urban area	0.77	0.79	-0.062	0.646
Decision making power	0.25	0.26	-0.011	0.836
Asset index	-0.42	-0.33	-0.038	0.312

1=Mean in near miss.

2=Mean in uncomplicated delivery.

3=Standardised difference.

4=t-test p-value of the difference in means between groups of women.

Table **5** shows the differences in household economic indicators between near miss and control groups with and without matching. Almost all outcome indicators had a negative sign, suggesting a persistent negative effect of the near miss event on households. However with matching, the effects became weaker and we had higher p-values, which indicates that part of the difference in outcomes is due to baseline differences between comparison groups.

**Table 5:** Comparison of household indicators between near-miss and the uncomplicated delivery groups.

		**(a)**		**(b)**	
	**Indicators**	**Coeff**	**p**	**Coeff**	**p**
**Food consumption^1^**	Rice consumption (days per week)	-0.582***	0.000	-0.362**	0.048
	Meat consumption (days per week)	-0.420**	0.019	-0.168	0.333
	Fish consumption (days per week)	-0.336*	0.058	-0.177	0.364
	Milk consumption (days per week)	-0.254	0.130	-0.231	0.238
	Total relatively expensive foods consumed a week	-1.593***	0.000	-0.939*	0.083
**Other economic indicators**	Income per capita	-2033.773	0.320	962.618	0.683
	Average expenditure per school-age child	-2.0e^+4^***	0.001	-1.6e+04***	0.001
	Number of children at school divided by school-age children	-0.077***	0.008	-0.049	0.265
	Number of times per week woman receives money for daily cooking	0.111***	0.001	0.061	0.121
	Woman has not eaten the day before	-0.015	0.263	-0.013	0.409
**Child development indicators**	Index child weight	-0.336	0.220	-0.259	0.409
	Index child height	-1.607***	0.005	-1.387***	0.007

Near miss vs. uncomplicated delivery women; 484 cases in control group and 214 in treatment group.

Note: Significance levels: *** p < 0.01; ** p < 0.05; * p < 0.10.

1=Relatively expensive food consumption

(a)=Unmatched case – control.

(b)=Matched case – control.

Coeff = coefficient .

p=p-value.

Looking at statistically significant coefficients after matching, we found that child development measured through the height of the index child was significantly affected, 4-5 years after their mothers were hit by the near miss event. We also found that households of near miss women spent significantly less money per school-age child and that they ate significantly less rice a week, up to 4-5 years after the near-miss event. We did not observe any statistically significant difference in total per capita expenditure between the treatment and comparison groups. Additionnally, no significant difference was observed on the proportion of school-age children who actually go to school or on the index child weight. However, we found at the 10% significance level that 4-5 years after the shock, households of near miss women consumed significantly less relatively expensive foods per week. 

Besides studying effects of severe obstetric complications on the consumption of relatively expensive food items, we investigated their effects on food security, as measured by the use of strategies to cope with foods shortages, strategies that were described by Maxwell in his paper in 1996 [41]. 

Table **6** shows a profusion of negative impacts, except for the strategy which consists of picking foods from the bush and for the overall food security index. Again, this may suggest a persistent negative effect of the near miss event on household food security. However, the evidence was not convincing as none of our indicators nor the index of food insecurity were significant after PSM at the conventional level of 5%.

**Table 6:** Comparison of food security/insecurity indicators between near-miss and the uncomplicated delivery groups.

	**(a)**		**(b)**	
**Indicators**	**Coeff**	**p**	**Coeff**	**p**
Eating non desired foods	-0.205**	0.018	-0.108	0.386
Asking help from relatives and friends	-0.106**	0.038	-0.025	0.678
Borrowing money or cereals	-0.057	0.183	-0.033	0.461
Buying foods in credit	-0.101***	0.008	-0.078	0.118
Picking foods from the bush	0.017	0.539	0.015	0.571
Limiting meals portion	-0.142**	0.021	-0.026	0.759
Reduction of household expenses^1^	-0.228**	0.023	-0.154	0.166
Woman limiting her meals portion^2^	-0.137**	0.027	-0.030	0.743
Woman reducing number of meals she eats/day	-0.106*	0.054	-0.024	0.738
Reducing number of meals taken by children	-0.069**	0.024	-0.060	0.102
Woman begging to feed her children^3^	-0.029**	0.015	-0.031	0.133
Woman skipping entire days without eating	-0.042**	0.037	-0.038	0.189
Children obliged to skip a day without eating	-0.014**	0.042	-0.012	0.203
Overall food insecurity index	1.214***	0.003	1.179	0.277

Near miss vs. uncomplicated delivery women; 484 cases in control group and 214 in treatment group.

1=Reduction of household expenses on other essential needs.

2=Woman limiting her meals portion to ensure enough foods for her kids.

3=Woman begging to feed her children and herself.

Note: Significance levels: *** p < 0.01; ** p < 0.05; * p < 0.10.

(a)=Unmatched case – control.

(b)=Matched case – control.

Coeff = coefficient.

p=p-value.

Table **7** shows differences between exposed and control groups on a series of quality of life indicators. Once more, many indicators appeared with a negative sign, even after matching, which may indicate a long term negative effect of the health shock on women’s quality of life. Focusing on statistically significant results, we found that women who were hit by the near miss event were, 4-5 years after the shock, significantly less satisfied with their health and with their perceived quality of life. Surprisingly, we also found at the 10% significance level that near miss women experienced less negative feelings about suicide, anxiety and depression in the month preceding the interview compared to women with uncomplicated deliveries. 

**Table 7:** Comparison of quality of life indicators between near-miss and the uncomplicated delivery group.

	**(a)**		**(b)**	
**Indicators (satisfaction with or appreciation of:)**	**Coeff**	**p**	**Coeff**	**p**
Her quality of life	-0.141***	0.007	-0.154***	0.003
Her health	-0.213***	0.000	-0.163**	0.036
The physical pain she feels	-0.093*	0.079	-0.066	0.274
Her daily need of drugs to carry out activities	-0.087**	0.036	-0.074	0.139
Her life	0.017	0.697	0.034	0.532
The meaning of her life	-0.067	0.113	-0.061	0.179
Her capacity to concentrate	-0.057	0.217	-0.040	0.539
Her own security	-0.115**	0.050	-0.077	0.249
The cleanliness of her environment	-0.054	0.294	-0.029	0.611
The energy she has in her daily life	-0.121**	0.013	-0.059	0.316
Her physical appearance	-0.044	0.373	0.005	0.931
Money availability for her daily life	-0.155***	0.005	-0.095	0.148
Her access to information in her daily life	-0.075	0.174	-0.042	0.492
Her capacity to find out time to relax	-0.044	0.440	0.038	0.561
Her capacity to move around	-0.111*	0.085	-0.015	0.840
Her sleeping quality	0.027	0.680	0.094	0.222
Her capacity to perform activities	-0.139***	0.005	-0.079	0.221
Her capacity to work	-0.138**	0.019	-0.093	0.242
Vis-à-vis herself	-0.079	0.125	-0.035	0.573
The relations she has with people	0.005	0.888	0.027	0.540
Her sexual life	-0.086	0.139	-0.043	0.561
Help she receives from relatives	-0.019	0.751	0.027	0.717
Her living conditions	-0.096	0.178	-0.067	0.374
Her access to health services	-0.077	0.101	0.007	0.989
Her mean of transport	-0.031	0.690	0.044	0.598
Experienced of negative feelings1	0.015	0.779	0.084*	0.092
Quality of life index	-0.076**	0.010	-0.032	0.313

Near miss vs. uncomplicated delivery women; 484 cases in control group and 214 in treatment group.

Negative feelings experienced by women during the last 4 weeks about suicide, anxiety and depression.

Note: Significance levels: *** p < 0.01; ** p < 0.05; * p < 0.10.

(a)=Unmatched case – control.

(b)=Matched case – control.

Coeff = coefficient.

p=p-value.

### Disaggregated analysis: the importance of pregnancy outcome

The near miss group consisted of women with different delivery outcomes including live birth, abortion and perinatal death. The long term outcomes for the near miss group may depend on the delivery outcome. For example, it is likely that giving birth to a live baby may incur extra costs (purchase of care, vaccinations, feeding etc) for women who are already in a distressing situation. To analyze the role of the pregnancy outcome, we disaggregated the near miss group into these three different groups and estimated the long term impact for each category separately. 

Table **8** presents results of the propensity score estimations for each type of near miss. **Model 1** which estimates the likelihood of a near miss event with live birth showed that economically active women, living in urban areas, were less likely to have a near miss complication with a live birth. On the contrary, single women, first polygamous married women or second or more than second polygamous married women were more likely to have a near miss with live birth. **Model 2** which estimates the likelihood of a near miss event with perinatal death showed that single women, first polygamous married women and women having a high number of pregnancies were more likely to have a near miss with perinatal death, while women with a high number of children still living with them were less likely to have a near miss with perinatal death. Finally, **Model 3** that estimates the likelihood of a near miss event with early pregnancy loss indicated that women living in urban or rural areas and who were autonomous in decision-making on household expenses were more likely to have a near miss complication with abortion. **Model 3** also showed that non-poor primary educated single women, women with a high number of living children with them were less likely to have a near miss with abortion. Table **9** further shows the balancing statistics of women’s characteristics based on standardised differences after matching. After matching, we found a satisfactory balance of the standardised differences of women’s characteristics in each model.

**Table 8:** Estimations of the propensity scores between each near-miss and control groups.

	**Model 1**		**Model 2**		**Model 3**	
**Variables**	**Coeff**	**SE**	**Coeff**	**SE**	**Coeff**	**SE**
Economic activity	-0.260*	0.132	-0.061	0.204	0.212	0.213
Married - monogamous	0.212	0.628	-	-	0.665	0.548
Polygamous - first wife	0.583*	0.347	0.966*	0.496	0.401	0.426
Polygamous - second or lower wife	0.907***	0.296	0.577	0.563	-0.004	0.406
Single	0.443**	0.208	0.726**	0.316	-0.426*	0.253
Number of live births still living with mother	-0.061	0.079	-0.311***	0.107	-0.136*	0.072
Number of pregnancies	0.050	0.055	0.284***	0.069	-	-
Modern primary education	-0.042	0.167	-0.319	0.280	-0.541*	0.320
Modern secondary education	0.046	0.178	-0.015	0.289	-0.244	0.307
Alphabetisation or ‘coranique’	0.102	0.236	0.102	0.236	-0.071	0.350
Living in a rural area	0.089	0.396	1.172	0.856	5.284***	0.441
Living in a urban area	-0.915***	0.353	0.099	0.826	4.887***	0.313
Decision making power	0.029	0.150	-0.168	0.253	0.494**	0.224
Asset index	-0.009	0.034	-0.023	0.057	-0.129**	0.064
Constant	-0.367	0.427	-2.528***	0.924	-6.082	0.000
N	590		493		496	
Pseudo R2	0.0654		0.1767		0.1709	
LR chi^2^	39.86		43.72		39.64	
Prob>chi^2^	0.0003		0.0000		0.0002	

Notes. Significance levels: *** p<0.01; ** p<0.05; * p<0.10. Balancing property satisfied for all models.

Model 1 = Near miss with live birth vs. uncomplicated delivery women. Model 2 = Near miss with perinatal death vs. uncomplicated delivery women. Model 3 = Near miss with abortion vs. uncomplated delivery women.

**Table 9:** Standardised differences after matching between each near-miss and control groups on the propensity score.

	**Model 1**				**Model 2**				**Model 3**			
**Variables**	**NM^1^**	**Un^2^**	**SD^3^**	**P^4^**	**NM^1^**	**Un^2^**	**SD^3^**	**P^4^**	**NM^1^**	**Un^2^**	**SD^3^**	**P^4^**
Economic activity	0.38	0.36	0.036	0.695	0.44	0.45	-0.015	0.938	0.65	0.61	0.072	0.719
Married - monogamous	0.01	0.01	-0.004	0.972	-	-	-	-	0.06	0.07	-0.053	0.701
Polygamous - first wife	0.06	0.05	0.017	0.863	0.09	0.09	-0.024	0.880	0.13	0.08	0.171	0.268
Polygamous - second or lower wife	0.13	0.09	0.139	0.124	0.03	0.01	0.085	0.711	0.10	0.09	0.015	0.944
Single	0.71	0.75	-0.093	0.353	0.79	0.77	0.050	0.807	0.42	0.51	-0.182	0.313
Number of live births still living with mother	2.13	2.12	0.005	0.960	2.53	2.47	0.033	0.842	2.19	2.34	-0.087	0.623
Number of pregnancies	2.95	2.90	0.022	0.824	4.50	4.43	0.028	0.861	-	-	-	-
Modern primary education	0.24	0.23	0.021	0.841	0.15	0.13	0.047	0.824	0.10	0.10	-0.016	0.933
Modern secondary education	0.30	0.31	-0.033	0.734	0.21	0.20	0.008	0.970	0.16	0.19	-0.060	0.775
Alphabetisation or ‘coranique’	0.08	0.08	-0.010	0.922	0.12	0.15	-0.118	0.970	0.10	0.09	0.024	0.909
Living in a rural area	0.16	0.13	0.083	0.174	0.26	0.27	-0.020	0.855	0.19	0.18	0.029	0.844
Living in a urban area	0.78	0.81	-0.091	0.038	0.71	0.69	0.039	0.740	0.81	0.82	-0.028	0.844
Decision making power	0.23	0.23	-0.004	0.971	0.21	0.18	0.052	0.718	0.39	0.41	-0.053	0.775
Asset index	-0.15	0.01	-0.072	0.464	-0.74	-0.59	-0.067	0.718	-1.15	-0.88	-0.137	0.433

Model 1 = Near miss with live birth vs. uncomplicated delivery women. Model 2 = Near miss with perinatal death vs. uncomplicated delivery women. Model 3 = Near miss with abortion vs. uncomplated delivery women.

1=Mean in near miss. 2=Mean in uncomplicated delivery. 3=Standardised difference. 4=t-test p-value of the difference in means between groups of women.

Table **10** presents differences in household economic indicators between each type of near miss and the control group. It is noteworthy that most indicators at household level in the three models appeared with a negative sign (with and without matching). Looking at statistically significant coefficients after matching, we found that the development of the surviving child of near miss women with live birth measured through his height was significantly lower than the one of the uncomplicated delivery child, up to 4-5 years after the health shock **Model 1(b**). We also found that 4-5 years after the shock, households of near miss women with live birth, in addition to consuming significantly less rice a week, spent significantly less money per school-age child **Model 1(b**). We were surprised to find that the number of times per week that women received money from their husband/partner for daily cooking seemed higher among near miss women with live birth compared to uncomplicated delivery women. However, this was not significant at the 5% level **Model 1(b**). **Model 2** in the same table which compares household economic indicators between near miss women with perinatal death and their controls indicated that near miss women with perinatal death were significantly less likely to consume relatively expensive foods in a week, and that their households were significantly likely to spend less money on children’s education compared to uncomplicated delivery households **Model 2(b**). Moreover, we found at the 10% significance level, that near miss women with perinatal death consumed significantly less meat, fish and milk, 4-5 years after the near miss event **Model 2(b**). However, we were surprised to find that near miss women with perinatal death were more likely to eat the day before the interview as compared to uncomplicated delivery women **Model 2(b**). **Model 3(b**) which presents the comparison of household level economic indicators between near miss women with abortion and their controls after matching showed that households of near miss women with abortion consumed significantly less milk a week, up to 4-5 years after the event. We also found at the 10% level, that they spent less money on children’s education. 

**Table 10:** Comparison of household indicators between each group of near-miss and the uncomplicated delivery group.

		**Model 1**				**Model 2**				**Model 3**				
		**(a)**		**(b)**		**(a)**		**(b)**		**(a)**			**(b)**	
	**Indicators**	**Coeff**	**p**	**Coeff**	**p**	**Coeff**	**p**	**Coeff**	**p**	**Coeff**	**p**	**Coeff**		**p**
**Food consumption**	Rice consumption^1^	-0.400***	0.032	-0.346**	0.050	-0.834**	0.011	-0.484	0.136	-1.045***	0.002	-0.388		0.240
	Meat consumption^1^	-0.247	0.244	-0.220	0.362	-0.736*	0.052	-0.552*	0.070	-0.787**	0.044	-0.350		0.309
	Fish consumption^1^	-0.159	0.451	-0.219	0.368	-1.007***	0.006	-0.650*	0.066	-0.320	0.404	0.006		0.989
	Milk consumption^1^	-0.032	0.874	-0.083	0.718	-0.630*	0.073	-0.505*	0.065	-0.742**	0.040	-0.594**		0.012
	Foods consumed^2^	-0.838	0.118	-0.868	0.121	-3.207***	0.001	-2.190**	0.015	-2.895***	0.003	-1.326		0.128
**Other economic**	Income per capita	528.666	0.831	1144.046	0.751	-4814.236	0.171	1704.44	0.581	-9e+03**	0.015	-3e+04		0.188
	Average expenditure^3^	-1.6e+05*	0.099	-2.3e+05**	0.015	-4e+05***	0.007	-2.4e+04**	0.014	-3e+04**	0.033	-1.3e+04*		0.061
	Schooling ratio^4^	-0.057	0.115	-0.062	0.104	-0.110**	0.022	-0.068	0.327	-0.084	0.114	-0.083		0.271
	Money cooking^5^	0.106***	0.004	0.084*	0.067	0.096	0.127	-0.051	0.591	0.148**	0.026	0.061		0.480
	Food availability^6^	-0.026	0.106	-0.027	0.224	0.022	0.386	0.013**	0.024	-0.011	0.695	-0.019		0.605
**Child development**	Index child weight	-0.336	0.224	-0.362	0.266	**-**	**-**	**-**	**-**	**-**	**-**	**-**		**-**
	Index child height	-1.512***	0.008	-1.590***	0.004	**-**	**-**	**-**	**-**	**-**	**-**	**-**		**-**

Near miss vs. uncomplicated delivery women; 484 cases in control group and 214 in treatment group.

Model 1 = Near miss with live birth vs. uncomplicated delivery women; 484 cases in control group and 127 in treatment group.

Model 2 = Near miss with perinatal death vs. uncomplicated delivery women; 484 cases in control group and 43 in treatment group.

Model 3 = Near miss with abortion vs. uncomplicated delivery women; 484 cases in control group and 44 in treatment group.

Note: Significance levels: *** p < 0.01; ** p < 0.05; * p < 0.10.

1=(days per week). 2=Total relatively expensive foods consumed. 3=Average expenditure per school age child. 4=Number of children at school divided by the number of school-age child. 5=Number of times per week woman receives money from husband/partner for daily cooking. 6=Woman has not eaten the day before the interview.

(a)=Unmatched case – control.

(b)=Matched case – control.

Coeff = coefficient.

p=p-value.

Table **11** presents the results of the long term effects of each type of near miss complication on household food security. Most of the indicators that measured food security with and without matching at household level appeared with a negative sign for all models. This may be a signal of persisting negative long term effect of the near miss event. Looking again at results after matching, we found a number of statistically significant indicators only in **model 3(b**). In particular, we found that near miss women with abortion were significantly likely to reduce the number of meals they eat per day. Their households were also more likely to buy food on credit compared to uncomplicated delivery households. We also found, at the 10% significance level, that near miss women with abortion were more likely to limit their meal portions to ensure more food for their children. In addition, they were also more likely to limit household members’ meal portions, to reduce household expenses on other essential needs and to eat non-desired foods, compared to uncomplicated delivery women. We were surprised to find at the 10% level that near miss women with abortion and their household were likely to experience more food security as measured by the food insecurity index compared to their counterparts.

**Table 11:** Comparison of food security/insecurity indicators between each group of near-miss and the uncomplicated delivery group.

	**Model 1**				**Model 2**				**Model 3**			
	**(a)**		**(b)**		**(a)**		**(b)**		**(a)**		**(b)**	
**Indicators**	**Coeff**	**p**	**Coeff**	**p**	**Coeff**	**p**	**Coeff**	**p**	**Coeff**	**p**	**Coeff**	**p**
Eating non desired foods	-0.095	0.338	-0.094	0.333	-0.479***	0.006	-0.393	0.072	-0.359**	0.048	-0.372*	0.057
Asking help from relatives and friends	-0.094	0.109	-0.080	0.281	-0.163	0.106	-0.120	0.402	-0.097	0.355	-0.130	0.238
Borrowing money or cereals	-0.036	0.461	-0.029	0.654	-0.073	0.391	-0.076	0.533	-0.125	0.162	-0.169	0.146
Buying foods in credit	-0.077*	0.069	-0.075	0.207	-0.020	0.777	-0.000	0.998	-0.288***	0.000	-0.295**	0.047
Picking foods from the bush	0.033	0.312	0.018	0.547	-0.023	0.705	0.028	0.481	2.67e-17	1.000	-0.031	0.653
Limiting meals portion	-0.075	0.280	-0.066	0.399	-0.234*	0.055	-0.145	0.281	-0.312**	0.017	-0.322*	0.097
Reduction of household expenses^1^	-0.138	0.234	-0.155	0.256	-0.418**	0.038	-0.333	0.183	-0.389*	0.064	-0.426*	0.078
Woman limiting her meals portion^2^	-0.084	0.236	-0.093	0.182	-0.221*	0.073	-0.134	0.356	-0.265**	0.045	-0.295*	0.098
Woman reducing number of meals she eats	-0.038	0.537	-0.044	0.470	-0.160	0.145	-0.075	0.610	-0.323***	0.006	-0.333**	0.036
Reducing number of meals taken by children	-0.068*	0.053	-0.054	0.284	-0.040	0.458	-0.033	0.483	-0.110*	0.077	-0.112	0.328
Woman begging to feed her children^3^	-0.038***	0.008	-0.040	0.149	-0.027**	0.016	-0.030	0.349	0.002	0.797	0.002	0.324
Woman skipping entire days without eating	-0.050**	0.032	-0.052	0.122	-0.037	0.272	-0.042	0.345	-0.011	0.753	-0.021	0.497
Children obliged to skip a day without eating	-0.006	0.319	-0.005	0.602	-0.057***	0.000	-0.058	0.196	0.002	0.797	0.002	0.310
Overall food insecurity index	0.759	0.105	1.492	0.159	1.953**	0.016	2.641	0.234	2.273***	0.008	4.915*	0.058

Near miss vs. uncomplicated delivery women; 484 cases in control group and 214 in treatment group.

Model 1 = Near miss with live birth vs. uncomplicated delivery women; 484 cases in control group and 127 in treatment group.

Model 2 = Near miss with perinatal death vs. uncomplicated delivery women; 484 cases in control group and 43 in treatment group.

Model 3 = Near miss with abortion vs. uncomplicated delivery women; 484 cases in control group and 44 in treatment group.

1=Reduction of household expenses on other essential needs. 2=Woman limiting her meals portion to ensure enough foods for her kids. 3=Woman begging to feed her children and herself.

Note: Significance levels: *** p < 0.01; ** p < 0.05; * p < 0.10.

(a)=Unmatched case – control.

(b)=Matched case – control.

Coeff = coefficient.

p=p-value.

Table **S1** presents the results of the long term effects of each type of near miss complication on women’s subjective quality of life. The appearance of the negative sign for most indicators with and without matching in **Model 1** which compares women’s quality of life between near miss women with live birth and their controls may suggest a negative impact. Looking at statistically significant results after matching, we found that near miss women with live birth were significantly less likely to be satisfied with their health and with their quality of life. They were aslo significantly less likely to appreciate the meaning of their life, 4/5 years after the near miss event compared to uncomplicated delivery women **Model 1(b**). In addition, we found at the 10% level that they were less likely to appreciate: the cleanliness of their environment, their access to information in their daily life, their living conditions and finally, less likely to be satisfied with their overall quality of life as measured through the quality of life index, up to 4-5 years after the near miss event **Model 1(b**). 

On the contrary, the occurrence of positive signs with and without matching when comparing women’s quality of life between near miss women with perinatal death and their controls (**Model 2**) and near miss women with abortion and their controls (**Model 3**) seems to indicate that the impact of the near miss event is evened out over time. In particular, we found after matching, that near miss women with perinatal death were significantly more likely to appreciate the meaning of their life and to experience less negative feelings about suicide, anxiety and depression in the month preceding the interview compared to women with uncomplicated delivery **Model 2(b**). Furthermore, we found, at the 10% significance level, that they were likely to be satisfied with their life, with their capacity to find time to relax and with their relations with people **Model 2(b**). We also found at the 10% significance level, that near miss women with abortion were likely to be satisfied with their relations with people **Model 3(b**)**.**

## Discussion and Conclusion

This study investigated the long term effects of near miss obstetric complications on the household economy and the well-being of women and their children in Burkina Faso. Although there is a growing literature on the consequences of near miss events, evidence on the long term economic consequences of life-threatening events is scarce. The contribution of this paper to the literature lies in the fact that it has a clinically-based and more consistent identification of near miss women than other studies. This identification was done prospectively in hospitals, allowing us to have a control on the cases we included. Moreover, it has a long run follow-up period – up to 5 years. This long follow-up is necessary to investigate the long term impact of severe maternal morbidity. Few studies have followed women as long as we did. In addition, we used a propensity score matching technique to deal with possible selection biases. 

The aggregated results of this study indicated that near miss events have a relatively small impact on near miss women as a whole group. We found evidence that near miss events durably affect the development of the index child measured through his or her height. Surprisingly, we did not find evidence that the near miss event affected food security. However, we found strong evidence of a negative association between the near miss event and the consumption of relatively expensive but essential foods such as rice, which is an important part of the population diet. We believe that the inability to ensure this essential consumption may reflect the overall resource shortages that Powell-Jackson & Hoque (2011) [43] mentioned in their study. We also found that households of near miss women experienced reduced investments in education. 

Moreover, in investigating the effects of near miss events on household food security, we examined food availability rather than food quality measured through calorie uptake, necessary to ensure healthy development. Therefore, we believe that one or more alternative factors may explain the negative effects we found on index child development and further research is needed here. We also found evidence that near miss events were associated with women’ dissatisfaction with their quality of life and perceived health. This is not surprising as some women ended up living with severe sequelae such as postpartum incontinence and fistulae [7]. 

The disaggragated results however showed that the near miss event had a significant negative impact for some sub-groups of near-miss women, on: the child’s development and education, education expenditures, relatively expensive food consumption, overall food security and women’s quality of life. Our study demonstrated that the nature of the pregnancy outcome determined differences in the results for the near miss groups. It also indicated that distinguishing between near miss outcomes remains a key issue for investigating the long term consequences of severe obstetric complications. 

Among the near miss women with live birth, for example, the study identified significant negative effects on children’s development and education. This effect could be explained by resource shortages at household level, also identified by Powell-Jackson & Hoque (2011) [43]. Resources shortages may have led to the observed reduced investments in children’s education, and an inability to ensure a varied and protein-rich diet containing rice, meat, fish or milk. Our findings showed that near miss women with live birth were less likely to eat rice, a relatively expensive food item in Burkina Faso, but one which is an important part of the local diet. We postulate that mothers’ nutritional status may have been compromised following the near-miss event, and they might not have been able to produce milk rich enough in nutrients necessary to ensure a normal development of the index child, especially during the early stage of life. This, in the long term, could have contributed to lower growth of the index child of near miss women. 

The study also showed strong evidence that the event may have a long lasting negative impact on the percieved health and quality of life of near miss women with live birth. These findings could be the result of loss and disruption in bodily integrity through injury, ongoing illness, loss of strength and stamina, and finally disruption of social identity and social stability as highlighted by qualitative research findings [22]. 

For near miss women with a perinatal death, we found evidence of negative impacts on their weekly consumption of relatively expensive foods and on their children’s education. Again, the effects we observed could be attributable to resource shortages that force households to reduce consumption of foods which may be important components of the local diet or to reduce investments in human capital. We also found indications that the event was associated with a reduction of consumption of meat, fish and milk. We were surprised to find that near miss women with perinatal death were likely to have eaten the day before the interview compared to uncomplicated delivery women. We also found, counter-intuitively, evidence that near miss women with perinatal death experienced less negative feelings about suicide, anxiety, depression in the month preceding the interview, and greater appreciation of the meaning of their life, compared to women who had an uncomplicated delivery. Moreover, we found indications that near miss women with perinatal death were satisfied with their life, their capacity to find time to relax and their relations with people. 

For near miss women who experienced an abortion, we found evidence that it had a negative impact on overall food security. We found particularly that near miss women with abortion were more likely to buy food on credit, to reduce the number of meals per day and their consumption of milk per week. There were also indications that near miss women with abortion were likely to limit their meal portions and that members of their households were likely to: eat non-desired foods, to limit their meal portions, to reduce household expenses and also spend less money on children’s education. This is in contrast with Storeng et al. (2010) [14], who found that the impact of the near miss event on household food insufficiency tends to vanish between 6-12 months. Surprisingly, we did not find differences in well-being between women who experienced an abortion and women who had an uncomplicated delivery. We were also surprised to find indications of a positive impact on the food insecurity index. We argue that the relatively small number of women who experienced a near miss complication with abortion in our sample may explain these findings. There remains a gap in understanding about the long term socio-economic consequences of abortion for women in this setting [44]. 

We did not find any significant difference between each of the near miss groups and the uncomplicated delivery group on the average expenditure per capita. This finding was consistent with what was found by Powell-Jackson & Hoque (2011) [43] in Bangladesh, who did not find any difference in per capita income between their comparison groups. However, we think that the reduction in expenditures for education, on other essential needs and in weekly consumption of relatively expensive foods could be the result of the resource shortages they mentioned in their findings [43]. 

The fact that we found few effects for the whole group of near miss women must not hide the importance of the findings for each near miss group. Policy actions that target pregnant women must take these differences between near miss groups into account. In particular, we think that fee exemptions for delivery care for indigent women are needed in order to better protect them from economic shocks and the subsequent economic difficulties. In Burkina Faso, the national subsidy policy for deliveries and emergency obstetric care actually specifies fee exemptions for indigent women [45], but research has demonstrated that the poorest women are rarely exempted [46], and that the exemption policy has never really been implemented [47]. The findings of this study reaffirm the need to make exemption policy work for indigent women. In this respect, results from recent studies conducted in Burkina Faso for the identification of indigents [48,49] could serve as a basis into implementing an exemption policy that works. 

## Supporting Information

Table S1 **Comparison of quality of life indicators between each group of near-miss and the uncomplicated delivery group.**
(DOC)

## References

[1] UN (2000) United Nations Millennium Development Declaration, General Assembly resolution, A/RES/55/2.

[2] Starrs AM (2006) Safe motherhood initiative: 20 years and counting. Lancet 368: 1130-1132. doi:10.1016/S0140-6736(06)69385-9. PubMed: 17011924. 17011924

[3] Hogan MC, Foreman KJ, Naghavi M, Ahn SY, Wang M et al. (2010) Maternal mortality for 181 countries, 1980-2008: a systematic analysis of progress towards Millennium Development Goal 5. Lancet 375: 1609-1623. doi:10.1016/S0140-6736(10)60518-1. PubMed: 20382417. 20382417

[4] Lozano R, Wang H, Foreman KJ, Rajaratnam JK, Naghavi M et al. (2011) Progress towards Millennium Development Goals 4 and 5 on maternal and child mortality: an updated systematic analysis. Lancet 378: 1139-1165. doi:10.1016/S0140-6736(11)61337-8. PubMed: 21937100. 21937100

[5] Bang RA, Bang AT, Reddy MH, Deshmukh MD, Baitule SB et al. (2004) Maternal morbidity during labour and the puerperium in rural homes and the need for medical attention: A prospective observational study in Gadchiroli, India. BJOG 111: 231-238. doi:10.1111/j.1471-0528.2004.00063.x. PubMed: 14961884. 14961884

[6] Bhatia JC, Cleland J (1996) Obstetric morbidity in south India: results from a community survey. Soc Sci Med 43: 1507-1516. doi:10.1016/0277-9536(96)00105-0. PubMed: 8923622. 8923622

[7] Filippi V, Ganaba R, Baggaley RF, Marshall T, Storeng KT et al. (2007) Health of women after severe obstetric complications in Burkina Faso: a longitudinal study. Lancet 370: 1329-1337. doi:10.1016/S0140-6736(07)61574-8. PubMed: 17933647. 17933647

[8] Fortney JA, Smith JB (1996) The base of the iceberg: prevalence and perceptions of maternal morbidity in four developing countries. The Maternal Morbidity Network Family Health International, Research Triangle Park.

[9] Fronczak N, Antelman G, Moran AC, Caulfield LE, Baqui AH (2005) Delivery-related complications and early postpartum morbidity in Dhaka, Bangladesh. Int J Gynecol Obstet 91: 271-278. doi:10.1016/j.ijgo.2005.09.006. PubMed: 16246344. 16246344

[10] Uzma A, Underwood P, Atkinson D, Thackrah R (1999) Postpartum health in a Dhaka slum. Soc Sci Med 48: 313-320. doi:10.1016/S0277-9536(98)00319-0. PubMed: 10077279. 10077279

[11] Wagner KS, Ronsmans C, Thomas SL, Calvert C, Adler A et al. (2012) Women who experience obstetric haemorrhage are at higher risk of anaemia, in both rich and poor countries. Trop Med Int Health, 17: 9–22. PubMed: 21955293. 21955293 10.1111/j.1365-3156.2011.02883.x

[12] Xu K, Evans DB, Kawabata K, Zeramdini R, Klavus J et al. (2003) Household catastrophic health expenditure: a multicountry analysis. Lancet 362: 111-117. doi:10.1016/S0140-6736(03)13861-5. PubMed: 12867110. 12867110

[13] Borghi J, Hanson K, Acquah CA, Ekanmian G, Filippi V et al. (2003) Costs of near-miss obstetric complications for women and their families in Benin and Ghana. Health Policy Plan 18: 383-390. doi:10.1093/heapol/czg046. PubMed: 14654514. 14654514

[14] Storeng KT, Baggaley RF, Ganaba R, Ouattara F, Akoum MS et al. (2008) Paying the price: the cost and consequences of emergency obstetric care in Burkina Faso. Soc Sci Med 66: 545-557. doi:10.1016/j.socscimed.2007.10.001. PubMed: 18061325. 18061325

[15] Ruger JP, Jamison DT, Bloom DE, Canning D (1998) Health and the Economy. In: Merson MH Black RE Mills AJ Internation public health: diseases, programs, systems and policies. second ed. Sudberry, Ma.: Jones and Bartlett Publishers.

[16] Krishna A (2010) One illness away: why people become poor and how they escape poverty. XVII. Oxford: Oxford University Press p. 229 s. p

[17] McIntyre D, Thiede M, Dahlgren G, Whitehead M (2006) What are the economic consequences for households of illness and of paying for health care in low- and middle-income country contexts? Soc Sci Med 62: 858-865. doi:10.1016/j.socscimed.2005.07.001. PubMed: 16099574. 16099574

[18] Russell S, Gilson L (2006) Are health services protecting the livelihoods the urban poor in Sri Lanka? Findings from two low-income areas of Colombo. Soc Sci Med 63: 1732-1744. doi:10.1016/j.socscimed.2006.04.017. PubMed: 16766105. 16766105

[19] Russell S (2004) The economic burden of illness for households in developing countries: a review of studies focusing on malaria, tuberculosis, and human immunodeficiency virus/acquired immunodeficiency syndrome. Am J Trop Med Hyg 71: 147-155. PubMed: 15331831. 15331831

[20] Russell S (2005) Illuminating cases: understanding the economic burden of illness through case study household research. Health Policy Plan 20: 277-289. doi:10.1093/heapol/czi035. PubMed: 16000367. 16000367

[21] Wagstaff A, van Doorslaer E (2003) Catastrophe and impoverishment in paying for health care: with applications to Vietnam 1993-1998. Health Econ 12: 921-934. doi:10.1002/hec.776. PubMed: 14601155. 14601155

[22] Storeng KT, Murray SF, Akoum MS, Ouattara F, Filippi V (2010) Beyond body counts: a qualitative study of lives and loss in Burkina Faso after 'near-miss' obstetric complications. Soc Sci Med 71: 1749-1756. doi:10.1016/j.socscimed.2010.03.056. PubMed: 20541307. 20541307

[23] Ridde V, Meessen B, Kouanda S (2011) Selective free health care in sub-Saharan Africa: an opportunity for strengthening health systems?. Sante Publique 23: 61-67. PubMed: 21786740. 21786740

[24] de la santé Ministère Burkina Faso (2010) Annuaires statistiques 2009. Burkina Faso.

[25] WHO (2009) World health statistics 2009. Geneva: World Health Organisation.

[26] Babalola S (2011) Maternal reasons for non-immunisation and partial immunisation in northern Nigeria. J Paediatr Child Health 47: 276-281. doi:10.1111/j.1440-1754.2010.01956.x. PubMed: 21244560. 21244560

[27] Gage AJ (2007) Barriers to the utilization of maternal health care in rural Mali. Soc Sci Med 65: 1666-1682. doi:10.1016/j.socscimed.2007.06.001. PubMed: 17643685. 17643685

[28] Gertler P, Rahman O, Feifer C, Ashley D (1993) Determinants of pregnancy outcomes and targeting of maternal health services in Jamaica. Soc Sci Med 37: 199-211. doi:10.1016/0277-9536(93)90455-D. PubMed: 8351534. 8351534

[29] Lantz PM (2001) Socioeconomic Status and Health Care. International Encyclopedia Soc Behav Sciences:: 14558-14562

[30] Zere E, Tumusiime P, Walker O, Kirigia J, Mwikisa C et al. (2010) Inequities in utilization of maternal health interventions in Namibia: implications for progress towards MDG 5 targets. Int J Equity Health 9: 16. doi:10.1186/1475-9276-9-16. PubMed: 20540793. 20540793 PMC2898738

[31] Rubin DB (1980) Bias Reduction Using Mahalanobis-Metric Matching. Biometrics 36: 293-298. doi:10.2307/2529981.

[32] Bloom DE, Jamison DT, Canning D, Ruger JP (2009) Health and the Economy. In: Merson M Black R Mills A, International public health: diseases, programs, systems, and policies, Second Edition, Chapter 13, Sudbury, MA: Jones and Bartlett Publishers , pp 601-648

[33] Dor A, Gertler P, Van Der Gaag J (1987) Non-price rationing and the choice of medical care providers in rural Cote d'Ivoire. J Health Econ 6: 291-304. doi:10.1016/0167-6296(87)90017-8. PubMed: 10285439. 10285439

[34] Elo IT (1992) Utilization of maternal health-care services in Peru: the role of women's education. Health Transit Rev 2: 49-69. PubMed: 10148665. 10148665

[35] Furuta M, Salway S (2006) Women's position within the household as a determinant of maternal health care use in Nepal. Int Fam Plan Perspect 32: 17-27. doi:10.1363/3201706. PubMed: 16723298. 16723298

[36] Glick P, Randretsa I, Razafindravonona J (2000) Education and health services in Madagascar: utilization patterns and demand determinants.

[37] Mrisho M, Schellenberg JA, Mushi AK, Obrist B, Mshinda H et al. (2007) Factors affecting home delivery in rural Tanzania. Trop Med Int Health 12: 862-872. doi:10.1111/j.1365-3156.2007.01855.x. PubMed: 17596254. 17596254

[38] Stephenson R, Baschieri A, Clements S, Hennink M, Madise N (2006) Contextual influences on the use of health facilities for childbirth in Africa. Am J Public Health 96: 84-93. doi:10.2105/AJPH.2004.057422. PubMed: 16317204. 16317204 PMC1470452

[39] Thaddeus S, Maine D (1994) Too far to walk: maternal mortality in context. Soc Sci Med 38: 1091-1110. doi:10.1016/0277-9536(94)90226-7. PubMed: 8042057. 8042057

[40] Woldemicael G, Tenkorang EY (2010) Women's autonomy and maternal health-seeking behavior in Ethiopia. Matern Child Health J 14: 988-998. doi:10.1007/s10995-009-0535-5. PubMed: 19882240. 19882240

[41] Maxwell DG (1996) Measuring food insecurity: The frequency and severity of "coping strategies". Food Policy 21: 291-303. doi:10.1016/0306-9192(96)00005-X.

[42] WHO (2004) The World health Organization Quality of Life (WHOQOL)-BREF 2004. World Health Organization.

[43] Powell-Jackson T, Hoque ME (2012) Economic consequences of maternal illness in rural Bangladesh. Health Econ, 21: 796–810. PubMed: 21557382. 21557382 10.1002/hec.1749

[44] Singh S (2010) Global consequences of unsafe abortion. Womens Health (Lond Engl) 6: 849-860. 10.2217/whe.10.70 21118043

[45] de la Santé Ministère Burkina Faso (2006) Stratégie nationale de subvention des accouchements et des soins obstétricaux et néonatals d'urgence au Burkina Faso. In: Famille DdlSdl, editor. Ouagadougou

[46] Ridde V, Kouanda S, Bado A, Bado N, Haddad S (2012) Reducing the medical cost of deliveries in Burkina Faso is good for everyone, including the poor. PLOS ONE 7: e33082. doi:10.1371/journal.pone.0033082. PubMed: 22427956. 22427956 PMC3299743

[47] Ridde V (2008) The problem of the worst-off is dealt with after all other issues The equity and health policy implementation gap in Burkina Faso. Social Science & Medicine 66: 1368-1378. 18248864 10.1016/j.socscimed.2007.10.026

[48] Ridde V, Sombie I (2012) Street-level workers' criteria for identifying indigents to be exempted from user fees in Burkina Faso. Trop Med Int Health 17: 782-791. doi:10.1111/j.1365-3156.2012.02991.x. PubMed: 22512433. 22512433

[49] Ridde V, Yaogo M, Kafando Y, Sanfo O, Coulibaly N et al. (2010) A community-based targeting approach to exempt the worst-off from user fees in Burkina Faso. J Epidemiol Community Health 64: 10-15. doi:10.1136/jech.2010.120956.26. PubMed: 19692724. 19692724

