# Supplementary material for: The Long Term Economic Impact of Severe Obstetric Complications for Women and Their Children in Burkina Faso
Source: PLoS One. 2013 Nov 5;8(11):e80010. doi: 10.1371/journal.pone.0080010 (PMC3818276; doi:10.1371/journal.pone.0080010)
Supplement: Table S1 — Comparison of quality of life indicators between each group of near-miss and the uncomplicated delivery group. (DOC) [file pone.0080010.s001.doc]

**Table S1: Comparison of quality of life indicators between each group of near-miss and the uncomplicated delivery group.**

|  | **Model 1** | | | | **Model 2** | | | | **Model 3** | | | |
| --- | --- | --- | --- | --- | --- | --- | --- | --- | --- | --- | --- | --- |
|  | **(a)** | | **(b)** | | **(a)** | | **(b)** | | **(a)** | | **(b)** | |
| **Indicators (women with; or appreciation of:)** | **Coeff** | **p** | **Coeff** | **p** | **Coeff** | **p** | **Coeff** | **p** | **Coeff** | **p** | **Coeff** | **p** |
| Her quality of life | -0.165*** | 0.007 | -0.156** | 0.023 | -0.130 | 0.213 | -0.071 | 0.636 | -0.057 | 0.595 | 0.006 | 0.959 |
| Her health | -0.247*** | 0.000 | -0.246*** | 0.004 | -0.157 | 0.151 | -0.059 | 0.701 | -0.139 | 0.221 | -0.116 | 0.424 |
| The physical pain she feels | -0.093 | 0.130 | -0.080 | 0.267 | -0.066 | 0.529 | -0.061 | 0.615 | -0.128 | 0.241 | -0.146 | 0.237 |
| Her daily need of drugs to carry out activities | -0.083* | 0.082 | -0.070 | 0.179 | -0.086 | 0.291 | -0.074 | 0.453 | -0.109 | 0.198 | -0.123 | 0.340 |
| Her life | -0.029 | 0.560 | 0.004 | 0.942 | 0.113 | 0.194 | 0.136* | 0.069 | 0.097 | 0.226 | 0.136 | 0.164 |
| The meaning of her life | -0.130*** | 0.009 | -0.134** | 0.022 | 0.108 | 0.201 | 0.226*** | 0.002 | -0.004 | 0.966 | 0.032 | 0.702 |
| Her capacity to concentrate | -0.012 | 0.823 | -0.009 | 0.884 | -0.226** | 0.019 | 0.006 | 0.974 | -0.055 | 0.570 | 0.024 | 0.789 |
| Her own security | -0.116* | 0.090 | -0.097 | 0.208 | -0.125 | 0.284 | -0.047 | 0.787 | -0.098 | 0.418 | -0.006 | 0.954 |
| The cleanliness of her environment | -0.089 | 0.143 | -0.108* | 0.060 | 0.038 | 0.720 | 0.086 | 0.486 | -0.014 | 0.901 | 0.082 | 0.455 |
| The energy she has in her daily life | -0.122** | 0.031 | -0.087 | 0.169 | -0.133 | 0.167 | 0.060 | 0.656 | -0.109 | 0.276 | -0.069 | 0.634 |
| Her physical appearance | -0.048 | 0.397 | -0.029 | 0.694 | 0.035 | 0.723 | 0.171 | 0.178 | -0.116 | 0.279 | 0.015 | 0.928 |
| Money availability for her daily life | -0.132** | 0.042 | -0.113 | 0.130 | -0.219* | 0.053 | -0.102 | 0.365 | -0.183 | 0.124 | -0.075 | 0.603 |
| Her access to information in her daily life | -0.108* | 0.094 | -0.129* | 0.071 | 0.121 | 0.279 | 0.129 | 0.289 | -0.154 | 0.186 | -0.138 | 0.218 |
| Her capacity to find out time to relax | -0.032 | 0.622 | -0.037 | 0.539 | 0.028 | 0.808 | 0.244* | 0.087 | -0.173 | 0.158 | -0.014 | 0.938 |
| Her capacity to move around | -0.054 | 0.476 | -0.050 | 0.570 | -0.326** | 0.014 | -0.202 | 0.247 | -0.115 | 0.399 | -0.004 | 0.975 |
| Her sleeping quality | 0.079 | 0.287 | 0.084 | 0.290 | -0.012 | 0.927 | 0.079 | 0.659 | -0.150 | 0.284 | -0.037 | 0.835 |
| Her capacity to perform activities | -0.120** | 0.034 | -0.092 | 0.178 | -0.145 | 0.132 | 0.056 | 0.701 | -0.212** | 0.038 | -0.105 | 0.459 |
| Her capacity to work | -0.151** | 0.028 | -0.126 | 0.128 | -0.126 | 0.242 | 0.039 | 0.806 | -0.109 | 0.370 | -0.002 | 0.989 |
| Vis-à-vis herself | -0.076 | 0.212 | -0.050 | 0.501 | -0.099 | 0.346 | -0.116 | 0.353 | -0.070 | 0.513 | 0.042 | 0.668 |
| The relations she has with people | -0.034 | 0.461 | -0.012 | 0.839 | 0.074 | 0.346 | 0.170* | 0.083 | 0.093 | 0.257 | 0.095* | 0.086 |
| Her sexual life | -0.080 | 0.232 | -0.055 | 0.476 | -0.052 | 0.656 | 0.037 | 0.756 | -0.148 | 0.227 | -0.117 | 0.496 |
| Help she receives from relatives | -0.025 | 0.725 | -0.020 | 0.782 | -0.037 | 0.759 | 0.008 | 0.957 | 0.020 | 0.874 | -0.024 | 0.866 |
| Her living conditions | -0.137* | 0.099 | -0.164* | 0.053 | -0.076 | 0.598 | -0.070 | 0.681 | 0.051 | 0.734 | -0.024 | 0.891 |
| Her access to health services | -0.124** | 0.024 | -0.113 | 0.133 | 0.013 | 0.893 | 0.167 | 0.186 | 0.018 | 0.854 | -0.041 | 0.659 |
| Her mean of transport | -0.032 | 0.724 | -0.008 | 0.924 | 0.005 | 0.974 | 0.184 | 0.397 | -0.071 | 0.665 | -0.064 | 0.682 |
| Experienced of negative feelings^1^ | -0.008 | 0.894 | 0.010 | 0.983 | 0.138 | 0.206 | 0.260** | 0.036 | -0.029 | 0.803 | 0.100 | 0.453 |
| Quality of life index | -0.083** | 0.016 | -0.072* | 0.057 | -0.051 | 0.377 | 0.048 | 0.526 | -0.075 | 0.214 | -0.022 | 0.775 |

Near miss vs uncomplicated delivery women; 484 cases in control group and 214 in treatment group.

Model 1 = near miss with live birth vs uncomplicated delivery women; 484 cases in control group and 127 in treatment group.

Model 2 = near miss with perinatal death vs uncomplicated delivery women; 484 cases in control group and 43 in treatment group.

Model 3 = near miss with abortion vs uncomplicated delivery women; 484 cases in control group and 44 in treatment group.

1 Negative feelings experienced by women during the last 4 weeks about suicide, anxiety and depression.

Note: Significance levels: *** p < 0.01; ** p < 0.05; * p < 0.10.

(a)=Unmatched case – control.

(b)=Stratified matched case – control.

Coeff = coefficient.

p=p-value.
